# The First Survey of Isolation and Molecular Typing of *Toxoplasma gondii* by Bioassay and PCR Method in BALB/c Mice in Camels (*Camelus dromedarius*) from Eastern Iran

**Published:** 2018

**Authors:** Amir TAVAKOLI KARESHK, Razieh TAVAKOLI OLIAEE, Hossein MAHMOUDVAND, Amir KEYHANI, Mohammad Ali MOHAMMADI, Mehdi BAMOROVAT, Mohammad Ali HAJHOSSEINI, Naser ZIA-ALI

**Affiliations:** 1. Infectious Diseases Research Center, Birjand University of Medical Sciences, Birjand, Iran; 2. Dept. of Medical Parasitology and Mycology, School of Medicine, Kerman University of Medical Sciences, Kerman, Iran; 3. Razi Herbal Medicines Research Center, Lorestan University of Medical Sciences, Khorammabad, Iran; 4. School of Veterinary Medicine, Kerman University of Medical Sciences, Kerman, Iran; 5. Research Center for Tropical and Infectious Diseases, Kerman University of Medical Sciences, Kerman, Iran

**Keywords:** *Toxoplasma gondii*, Isolation, Bioassay, Genotyping, PCR-RFLP

## Abstract

**Background:**

We isolated *Toxoplasma gondii* from camels by bioassay method in mice model and detect parasitic DNA in brain mice by molecular methods.

**Methods:**

One hundred tissue samples including heart (n=50), and diaphragm (n=50) were collected from camels (n=50) slaughtered in abattoirs from Feb to Oct 2015 in three provinces located in eastern Iran. In first, blood sample from 50 camels was assayed for anti-*Toxoplasma* antibodies by modified agglutination test (MAT) test. Bioassay method was done in positive MAT blood camels in BALB/c mice and Nested PCR performed in seropositive tissue samples to amplify the B1 and GRA6 genes. The existence of polymorphic restriction sites for endonuclease MseI was used with PCRRFLP method and Sequencing analysis to evaluate the prevalence of type strains (I, II and III)

**Results:**

Overall, 13 (26%) of camels were positive with titer of 1:20 for toxoplasmosis and 13(26%) tissue samples of camels were found positive for the *T. gondii* B1 gene, including 7(14%) diaphragm, 6(12%) heart. Moreover, 3(6%) tissue samples of camels were found positive with GRA6 gene for *T. gondii*. There are three genotypes and mix genotype using MseI enzyme among all positive samples.

**Conclusion:**

The obtained results from serological and molecular tests demonstrated the infection of *T. gondii* with previously recognized genotypes in the tissues of camels for first time from Iran. Since consumption of meat camels are raising in Iran, there may be a high risk of toxoplasmosis through consumption of products from these hosts due to their susceptibility to the infection.

## Introduction

Toxoplasmosis, caused by the obligate intracellular, parasitic protozoan *Toxoplasma gondii*, is one of the most common parasitic infections of human and a wide spectrum of warm-blooded animals. It has been found world-wide from Alaska to Australia (1). Approximately one-third of world population have been infected with this parasite. Cats, both wild and domestic, are the only definitive hosts for *T. gondii*; whereas a considerable number of other homoeothermic animals, as well as human, are intermediate hosts (2).

Carnivores including human can normally become infected when they eat raw or under-cooked tissues containing tissue cysts or, occasionally, tachyzoites. Both herbivores and carnivores may ingest infective oocysts in food or water (2). Moreover, *T. gondii* can cross the placenta in some species, particularly sheep, goats, humans and small rodents.

In human, the infection does not cause serious symptoms, but in immune-compromised people toxoplasmosis can lead to serious symptoms including retinochoroiditis, lymphadenopathy, myocarditis, and encephalitis. In animals, toxoplasmosis causes considerable reproductive and economic problems as well as public health concerns as consumption of contaminated meat and milk damage the human health and make zoonotic transmission easy (3,4).

Camel meat is frequently consumed and is highly susceptible to the exposure of toxoplasmosis which may become the possible source of infection for the customers. Prevalence of *T. gondii* in camels is significant because of the continuous contamination of pastures by *T. gondii* oocysts which makes this parasite a common infectious agent among these animals (4,5). In Iran, the seroprevalence of toxoplasmosis was reported between 13%–35% for sheep, 13%–30% for goats, 0%–16% for cattle, 4%–10% for buffaloes and 6.6%–14.5% for camels in different parts of Iran (6–11). Previous studies with multilocus restriction fragment length polymorphism (PCR-RFLP) or microsatellite markers have reported that *T. gondii* has a clonal population structure consisting of three genetic lineages i.e., Type I, Type II and Type III (12). Reviews have shown a predominance of geno-type II in domestic animals of various parts of the world including France, Spain, Switzerland, Germany, and Iran (8–11). However, there is no study on genetic characterization of *T. gondii* isolates in domestic camels in Iran.

The present study was designed for detecting parasitic DNA in tissues from camels raised and slaughtered in eastern Iran as well as to genetically characterize infecting strains of *T. gondii*.

## Materials and Methods

### Sample collection

Overall, 100 tissue samples including heart (n=50) and diaphragm (n=50) as well as 50 blood samples were obtained from camels (n=50) slaughtered in abattoirs from three provinces located in eastern Iran including Kerman, Razavi Khorasan, and south Khorasan Provinces, Iran between Feb to Oct 2015. All the collected animals had been born and raised in southeastern Iran and were intended for human consumption.

### Serologic evaluation

All blood samples from camels were examined for *T. gondii* infection using the Modified agglutination test (Toxo screen DA, biomerieux®, France) (13). Sera were assayed at dilutions of 1/20 and 1/4000; a titer of 1:20 was considered positive and the tissue sample from seropositive animals was assayed for bioassay method.

### Bioassay of tissue samples for T. gondii

Approximately 50 gr of heart and diaphragm tissue samples from modified agglutination test (MAT) positive camels were processed for isolation of *T. gondii* (4). Briefly, each sample was cut in small pieces of approximately 1 cm^3^, homogenized in a blender for 30 sec, followed by suspension in 125 ml of saline solution (0.14 M NaCl) for another 30 sec. After homogenization, 250 ml of a pepsin solution (Merck KG.A, Darmstadt, Germany) was added. After an incubation of 1 h at 37 °C, the homogenate was filtered through two layers of gauze and centrifuged at 1200×g for 10 min. The supernatant was discarded and the pellet was resuspended in 15 to 20 ml of 1.2% sodium bicarbonate solution (pH=8.3) and recentrifuged at 1200×g for 10 min. The supernatant was discarded and the sediment was re-suspended in 5 to 10 ml of antibiotic saline solution (1000 U/ml penicillin and 100 μg streptomycin/ml in saline solution). One ml of this suspension was inoculated intraperitoneally (IP) in five BALB/c mice per sample (4).

Bioassays were performed within 1 to 3 d after the slaughter of the animals. The mice used were *T. gondii* seronegative female BALB/C mice, obtained from the animal facility of the Kerman University of Medical Sciences (Kerman, Iran). Non-infected mice (n=5) were kept separately as negative controls. The mice were given commercial pelleted feed and municipal chlorinated water ad libitum. The inoculated mice were observed daily for the presence of clinical signs until day 60 post inoculation. A *T. gondii* isolate was considered virulent if mortality of mice was observed within two weeks of infection (14). The mice were bled on day 45 post-inoculation and their sera tested for *T. gondii*-antibodies by the modified agglutination test (MAT).

Two months after intraperitoneal (IP) inoculation, the brain from surviving mice was removed after euthanasia with di-ethyl ether. Each brain was homogenized in 1 ml PBS (pH 7.2) using a mortar and pestle. Following microscopic examination and counting of cysts, the homogenates were stored in 1.5 ml Eppendorf tubes and frozen at −20 °C until DNA extraction was done. The number of cysts in three aliquots of 10 μl each was counted under a light microscope with a 100x magnification. The total number of cysts in the brain of each mouse was determined by converting the sum of cysts in 30 μl to the whole volume of the brain homogenates (15,16). A bioassay was considered positive if *T. gondii* cysts were detected in any of the five inoculated mice.

### DNA extraction

DNA was extracted from mice brain (17). Using the QIAamp Tissue kit (Qiagen). Briefly, 75–100 μl of homogenized brain (approximately 25 mg brain tissue), 180 μl of lysis buffer of the kit (ATL) and 20 μl of proteinase K were added and incubated at 56 °C until the tissue was completely lysed (60 to 90 min). The lysate was then mixed with 200 μl AL buffer and incubated for another 10 min at 70 °C. DNA was precipitated by addition of 200 μl ethanol (96%–100%). Then, the mixture was carefully applied to the QIAamp Mini spin column and centrifuged at 6000×g for 1 min. The columns were then washed by centrifugation using buffers AW1 and AW2, according to the manufacturer’s instruction. Finally, the DNA was eluted from the column using 50 μl of the elution buffer of the kit (AE) and stored at −20°C until used for genotyping.

### Ethical issues

This research project was approved by the Ethics Committee of the Kerman University of Medical Science. All efforts were made to minimize animal suffering during the course of the study (No. 94/389).

### Nested-PCR for B1 gene

Nested-PCR was performed in a final volume of 25 μl using the Taq DNA Pol 2.0 × master mix with an MgCl_2_ concentration adjusted to 1.5 mM; 100 ng/μl of DNA was used for the PCR assay. Each sample was tested in duplicate for each method. The nucleotide sequences of the primers for the nested-PCR assays targeting the B1 and GRA6 gene are listed in Table 1 (18, 19). The Nested-PCR assays were performed based on two repeated genomic targets, B1, to detect *T. gondii* DNA. The first round of PCR amplification contained 10 mMTris–HCl, pH 8.3 (at 25 °C), 50 mMKCl, 1.5 mM MgCl_2_, 5 μM each primer (Table 1), 250 μM each dNTP, 0.1 U *Taq* DNA polymerase, and 100 ng/μl(lμl) of extracted DNA. Reactions were started with an initial denaturation at 94 °C for 5 min and then cycled 30 times with denaturation at 94 °C for 20 sec, followed by annealing at 53 °C for 20 sec for B1 gene and finally an extension step at 72 °C for 20 sec followed by a 5 min final extension at 72 °C. A PCR negative-control sample omitted template DNA, replaced by sterile water and a positive control sample that used extracted DNA from *T. gondii* tachyzoites RH-strain.

**Table 1:** Primers for the nested-PCR assays targeting the B1 and GRA6 used for *T. gondii* molecular diagnosis

***Assay***	***Oligonucleotide sequence***	***Nucleotide positions***
B1-nested PCR	5′TCAAGCAGCGTATTGTCGAG	663–682
	5′CCGCAGCGACTTCTATCTCT	949–930
	5′GGAACTGCATCCGTTCATGAG	694–714
	5′-TCTTTAAAGCGTTCGTGGTC	887–868
Gra6 nested PCR	5′-GGCAAACAAAACGAAGTG-3′	223–240
	5′-CGACTACAAGACATAGAGTG-3′	1166–1147
	5′- GTAGCGTGCTTGTTGGCGAC-3′	372–391
	5′- TACAAGACATAGAGTGCCCC-3′	1162–1143

### Nested PCR for GRA6 gene

Nested PCR was done to amplify the coding region of the GRA6 gene. PCR amplification was performed with 1 μl of DNA template in 50 μl of a reaction mixture containing 10 mMTris-HCl, pH 8.3; 50 mMKCl; 2 mM MgCl2; 200 μM dNTPs; 0.5 μM each of oligonucleotide primers; 1.25 units of Taq DNA polymerase (Takara, Japan); and 50 pmol of each primer. All PCRs were performed in a FlexCycler (Analytik Jena, Germany). The first step of amplification was 5 min of denaturation at 94 °C. This step was followed by 35 cycles, with 1 cycle consisting of 30 sec at 94 °C, 60 sec at the annealing temperature for each pair of primers, and 90 sec at 72 °C. The final cycle was followed by an extension step of 7 min at 72 °C. The PCR primer pair was designed from the GRA6 gene sequence (20), GRA6FO:5′-GGCAAACAAAACGAAGTG-3′ and 1106 S. GRA6RO: 5′-CGACTACAAGACATAGAGTG-3′ (positions 223–240 and 1166–1147, respectively), used in the initial PCR reaction at an annealing temperature of 54 °C. The resulting amplification products were diluted to 1/10 in distilled water, and a second amplification was performed with the internal primers (21), GRA6R: 5′-GTAGCGTGCTTGTTGGCGAC3′ and GRA6; 5′TACAAGACATAGAGTGCCCC-3′ (positions 372–391 and 1162–1143, respectively), using 1 μl of the diluted product as the template at an annealing temperature of 60 °C. Five μlof PCR product was analyzed by electrophoresis in 1% agarose gel in Tris-acetate-EDTA buffer (TAE, pH 8), and visualized under UV light by ethidium bromide staining (0.5 *μ*g/ml; Applichem, Biochemica, Germany). The size of PCR target is expected to yield 791 bp for Gra6 positive reactions.

### PCR-RFLP

The GRA6 gene amplified product was exposed with *MseI* restriction endonuclease (10 U/*μ*l, 300 units), (Fermentas, Thermo Scientific, USA) for digestion. Briefly, 15 ml of PCR product was digested using 1.5 U of *MseI*enzyme and 2 U buffer R and incubated at 65 °C for 4 h in accordance with the manufacturer’s protocol. The restriction fragments were separated by electrophoresis in 2% agarose gel followed by staining with ethidium bromide and visualization under UV. The cut position of *MseI* in GRA6 genes of types I, II, and III was 168 bp and 712 bp, 71 bp and 694 bp, and 71 bp, 168 bp, and 712 bp, respectively (22).

### Sequencing

Sequence analysis for comparison of B1 and GRA6 genetic profiles were performed to determine type of *T. gondii* (I, II, III). Nested PCR products and internal primers were sent to Macrogene Company of South Korea. Results were aligned with BioEdit and sequence Scanner program and compared to the following sequence data available from GeneBank: AB235428, AB235433, AF239283 RH type I, AF239284, AB235430 Beverley type II and AF239286, AB235429NED type III, (GenBank). The phylogenetic relationships among genotypes were estimated using Maximum-likelihood analysis. Mega 6 software was also used to construct the phylogeny tree to compare our isolates with types submitted in Genebank. *Hammondia hammondi* (GenBank accession numbers: XM 008888391) was employed as the outgroup to root the resulting trees.

## Results

### MAT serologic test

Among 50 blood sample of camels, 13 (26%) were positive. a titer of 1:20 was considered positive and the tissue samples of positive isolates were collected for bioassay method.

### Nested PCR positive isolates

**Fig. 1:**
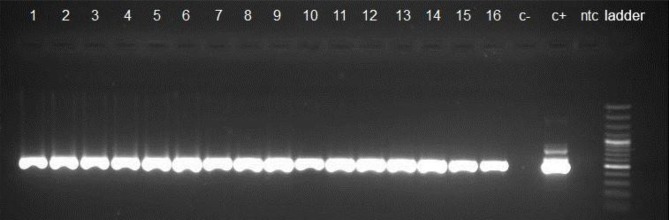
Agarose gel separation of representative nested PCR products of the B1 gene. Lane 1–16, positive isolates; c−: negative control, c+: positive control, ntc: negative sample, DNA ladder 100 bp

**Fig. 2:**
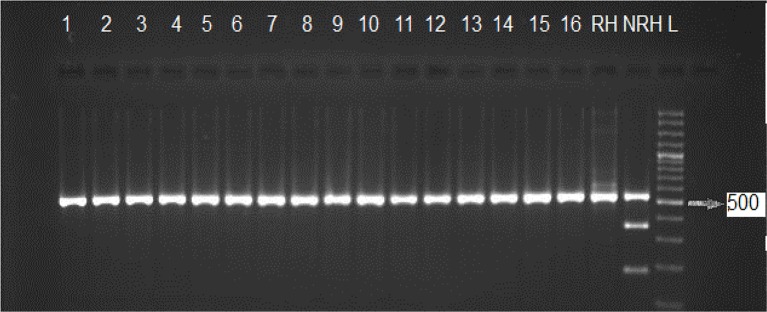
DNA fragment patterns of isolates after digestion of B1 gene with *Pml* enzyme. lanes 1–16 and RH type I, lane NRH type II or III., L 100 bp DNA ladder

Figures 1 and 2 indicate gel electrophoresis after PCR amplification with B1 primers and GRA6 respectively. Table 2 shows PCR results in the 100 tissue samples obtained by inoculating BALB/c mice including heart (n=50) and diaphragm (n=50) obtained from camel (n=50) slaughtered in abattoirs from eastern Iran. Totally, 13(26%) tissue samples of camels were found positive for the *T. gondii* B1 gene, including 7 (14%) diaphragms, and 6(12%) hearts. Among positive samples, 9(18%) and 4(8%) were male and female respectively.

**Table 2:** The result of *T. gondii* detection of camel’s sample by using PCR in inoculated mice with seropositive tissue of camels

***Tissue of the camels***	***N***	***PCR by B1 gene***	***PCR by Gra6 gene***
Heart	50	6 (12%)	1 (2%)
Diaphragm	50	7 (14%)	2 (4%)
Total positive	100	13 (26%)	3 (6%)

Only 3(6%) tissue samples of camels were found positive with GRA6 gene for *T. gondii*, including 2(4%) diaphragms and 1(2%) heart, of which, 2(4%) were male and 1(1%) was female. In the present study, total prevalence with the main marker of diagnosis in tissues, B1 gene, was 26% (Table 2).

### Genetic characterization by PCR-RFLP

**Fig. 3:**
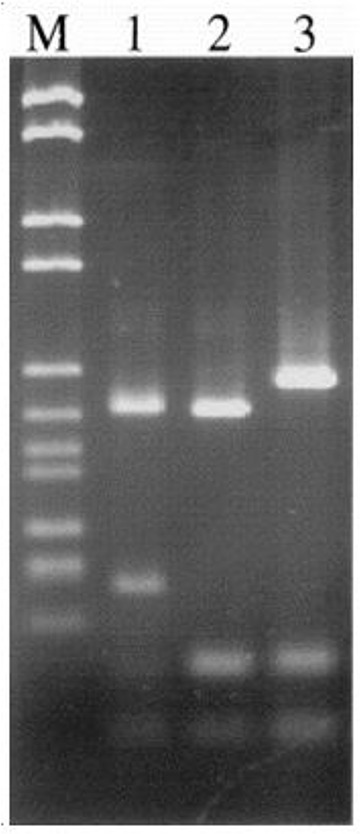
DNA fragment patterns of isolates after digestion of GRA6 gene with *MseI* enzyme. M, 100 bp DNA ladder. lane1–3 are *T. gondii* type I, II, III respectively

To determine the genetic characterization of isolates, genotyping of positive samples was examined by PCR-RFLP with GRA6 gene. As shown in Table 3, there were three genotypes and mix genotype using *MseI* enzyme among all positive samples (Fig. 3).

**Table 3:** Frequency and percent of the *T. gondii* genotypes in camel tissues by PCR-RFLP on GRA6 gene

***Animal***	***Genotype I /II, III***	***Genotype II***	***Genotype III***	***Genotype II/III***
Camel	7(14%)	1(2%)	-	5(10%)

### Sequencing and phylogenetic analysis

**Fig. 4:**
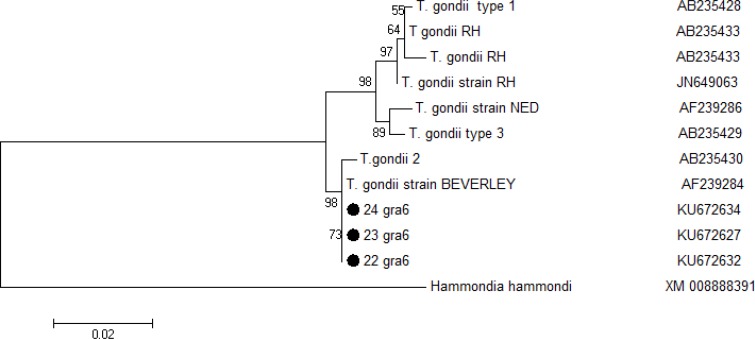
Phylogenetic tree of camel isolates, based on the GRA6 gene. The three sequence types identified in this study are shown. The maximum-likelihood analysis was used to construct the tree. The scale bar indicates distance. Nodal support is given as *P-*value

The phylogenetic analysis demonstrated that three samples 22, 23 and 24 were identified similar to type 2 with high similarity in sister clade (Fig. 4). Representative nucleotide sequences obtained in this study had similarity to type II and were deposited in the GenBank database under the accession numbers: KU672632, KU672636, KU672641, KU672645, and KU672652.

## Discussion

*T. gondii* infects nearly one-third of the world population. Although toxoplasmosis is asymptomatic in immunocompromised individuals, the disease is life-threatening during pregnancy and in immunocompromised drones (2).

Normally, humans become infected with *T. gondii* mainly via ingesting raw or undercooked meat of infected animals or by ingesting food or water contaminated with oocysts and the parasite can be transmitted to the fetus by the passage of tachyzoites through the placenta (1). Camels are the main livestock animals, particularly in developing countries such as Iran, and their products including meat and milk are widely used as important sources of food for humans around the world. The presence of *T. gondii* in milk (23) and tissues (24) of camels represents the likelihood of transmission to humans; mainly in rural communities in which raw milk and raw meat, can be commonly consumed (25). Camels can be infected by eating sporulated oocysts which are shed by definitive hosts such as cat in the surroundings (4).

The prevalence of *T. gondii* infection in camels varies depending on the geographical situation (26). In Iran, this prevalence ranges from 3.12% (27) to 90.90% in Turkey (28). A direct agglutination test (DAT) and indirect enzyme-linked immunosorbent assay (ELISA) kits were used to estimate the seroprevalence of *T. gondii* infection from camels in Ethiopia. An overall prevalence of 49.62% by DAT and 40.49% by indirect ELISA test were detected (29). Another study showed 12.6% seroprevalence of *T. gondii* infection among childbearing age women in Kerman city used by ELISA method (30). Recently study that performed in kerman provice by molecular methods with tavakoli kareshk et al shown 56.66% sheeps and 44.16% from goats were found to be positive for *T. gondii* B1 gene (31).Previous studies were performed on seroprevalence of toxoplasmosis in camels of Iran, for example, 6.60% of camels in Chaharmahal and Bakhtiari Province were positive for *T. gondii* antibodies (24). Another study demonstrated prevalence of *T. gondii* in 3.12% of milk of camels in Iran (27). However, to the best of our knowledge, there is no molecular study on *T. gondii* among camels in Iran. Therefore, this study for the first time was performed on isolation and molecular detection of *T. gondii* DNA in camels in Iran. In our study, all 13 seropositive samples with MAT test were also identified positive after bioassay by amplification of B1 gene on mice brain and not seen brain cysts of *T. gongii* by microscopy examination in inoculated BALB/c mice. The nested PCR method shows the high sensitivity and specificity for the detection of toxoplasmosis; furthermore, B1 gene because of having higher copies in genome of *T. gondii* and also high specificity and sensitivity is as suitable instrument to identify *T. gondii* (32). On the other hand, PCR-RFLP is considered as an appropriate technique to identify the genotypes of *T. gondii* isolates using analysis of the pattern derived from the cleavage of its DNA (17). Here, we found that 26% and 6% of camels in eastern Iran were infected with *T. gondii* by B1 and GRA6 genes, respectively. In this study to determine the genotyping of *T. gondii* isolates, GRA6 products were digested with *MseI* restriction enzyme, and the type of samples was determined by digestion patterns. There are three genotypes and mix genotype using *MseI* enzymes among all positive isolates.

In Iran, type II and III were found in 4 isolates from sheep according to microsatellite and GRA6 gene sequence analysis (33). Moreover, other studies reported type I in some domestic animals using PCR-RFLP based on GRA6 gene in Iran (34). The GRA6 sequencing and phylogenetic analysis confirmed that three samples 22, 23 and 24 were identified similar to type 2 with high similarity in sister clade.

## Conclusion

The findings of this study demonstrated the presence of *T. gondii* DNA in the tissues of camels from Iran for the first time. Since camels are the most important food sources in Iran, there may be a high risk of contamination through consumption of products from these hosts due to their susceptibility to the infection. Therefore, control of the infection in food animals is important for consumer protection.
